# Against All Odds: Genocidal Trauma Is Associated with Longer Life-Expectancy of the Survivors

**DOI:** 10.1371/journal.pone.0069179

**Published:** 2013-07-24

**Authors:** Abraham Sagi-Schwartz, Marian J. Bakermans-Kranenburg, Shai Linn, Marinus H. van IJzendoorn

**Affiliations:** 1 Center for the Study of Child Development and Department of Psychology, University of Haifa, Haifa, Israel; 2 Centre for Child and Family Studies, Rommert Casimir Institute for Developmental Psychopathology, Leiden University, AK Leiden, The Netherlands; 3 School of Public Health, University of Haifa, Haifa, Israel, and Unit of Clinical Epidemiology, Rambam Medical Center, Haifa, Israel; Swiss Tropical & Public Health Institute, Switzerland

## Abstract

Does surviving genocidal experiences, like the Holocaust, lead to shorter life-expectancy? Such an effect is conceivable given that most survivors not only suffered psychosocial trauma but also malnutrition, restriction in hygienic and sanitary facilities, and lack of preventive medical and health services, with potentially damaging effects for later health and life-expectancy. We explored whether genocidal survivors have a higher risk to die younger than comparisons without such background. This is the first population-based retrospective cohort study of the Holocaust, based on the entire population of immigrants from Poland to Israel (*N* = 55,220), 4–20 years old when the World War II started (1939), immigrating to Israel either between 1945 and 1950 (*Holocaust group*) or before 1939 (*comparison group*; not exposed to the Holocaust). Hazard of death – a long-term outcome of surviving genocidal trauma – was derived from the population-wide official data base of the National Insurance Institute of Israel. Cox regression yielded a significant hazard ratio (HR = 0.935, CI (95%) = 0.910–0.960), suggesting that the risk of death was reduced by 6.5 months for Holocaust survivors compared to non-Holocaust comparisons. The lower hazard was most substantial in males who were aged 10–15 (HR = 0.900, CI (95%) = 0.842–0.962, i.e., reduced by 10 months) or 16–20 years at the onset of the Holocaust (HR = 0.820, CI (95%) = 0.782–0.859, i.e., reduced by18 months). We found that against all odds genocidal survivors were likely to live longer. We suggest two explanations: Differential mortality during the Holocaust and “Posttraumatic Growth” associated with protective factors in Holocaust survivors or in their environment after World War II.

## Introduction

Does surviving genocidal experiences, like the Holocaust, lead to shorter life-expectancy? Such an effect is conceivable given that most survivors not only suffered psychosocial trauma such as witnessing violence and experiencing separation and loss of loved ones but they were also exposed to malnutrition and famine, restriction in hygienic and sanitary facilities, and lack of preventive medical and health services. One of the potential mechanisms for the association between early trauma and later disease morbidity and mortality is telomere erosion. Telomeres are the repetitive non-coding sequences at the end of chromosomes that function to cap the ends of chromosomes from the DNA damage [1]. They become shorter with each cell division and may be considered a biological marker for aging. Adverse events such as childhood maltreatment, neglect, and witnessing of violence may accelerate the process of telomere erosion, as documented in recent studies on severe childhood adversities [2], cumulative exposure to childhood violence [3], and the effects of low-quality institutional settings [4]. Thus, it is plausible to expect that Holocaust survivors have a higher risk to die younger than comparisons without Holocaust background. Surprisingly we show below that, against all odds, survivors are likely to live longer.

The most comprehensive study on genocidal survivors is a set of meta-analyses (71 samples; 12,746 participants), suggesting that Holocaust survivors are less well-adjusted than counterparts without Holocaust background, showing particularly more posttraumatic stress symptoms. [5] Their functioning in the domains of physical health and cognition however was not different from that of their comparisons.

The effect of genocidal experiences on death rates of the survivors is as yet unknown. Results of studies linking Holocaust traumatic stress to morbidity risk and death rates are inconclusive. Most studies are community-based, relying on small samples, self-report data, or records obtained from clinics. [6] The only study based on national records focused on various types of cancer, [7] and showed that the risk of particularly breast and colorectal cancer was somewhat higher among those exposed to the Holocaust than among those not exposed.

Here we report on the first retrospective population-based cohort study examining age of death among Holocaust survivors compared to peers without Holocaust background. Given the damaging living conditions of Holocaust survivors during part of their lives, we expected to find that the risk of Holocaust survivors to die younger is greater than that of comparisons without Holocaust background.

## Methods

We obtained our data from the entire population-wide official data set of the National Insurance Institute of Israel (NII). NII oversees various social security related issues, among which disability and aging. As such NII has data on all Israeli citizens regarding dates of death since 1948 (the year the State of Israel was founded). Israeli death registry regulations enforce any death to be immediately reported to the population registry and NII. Thus every death occurrence is reported in real time immediately after death by the nation-wide hospital and medical system and by the burial authority, which is administered nationally by the Ministry of Religious Services. The Committee for Information Transfer approved access to the data used in this paper (with concealed official IDs and names). The data was provided to the researchers in 2011.

We constructed two groups – Holocaust survivors and comparisons – consisting of immigrants to Israel from Poland, born between 1919 and 1935, and immigrating to Israel (until May 1948 the British Mandate of Palestine), either between 1945 and 1950 (Holocaust survivors) or before 1939 (comparisons). The working assumption in Holocaust research is that Jews living in Europe during the years of the war, regardless of direct experience (i.e. camps, hiding), should be defined as Holocaust survivors whereas those who left Europe before the war are non-Holocaust survivors.^1^ The year 1950 was chosen as an end point because census data showed that most survivors immigrated to Israel during 1945–1950 and we wanted to concentrate on the bulk of survivors who came with the massive immigration that followed the Holocaust.

The final cohort consisted of *n* = 55,220 participants, 41,454 and 13,766 cases in the Holocaust and comparison group, respectively. The *Holocaust group* consisted of immigrants to Israel who were 4–20 years old when World War II broke out. Similar to other studies, [5] it was assumed that any Jew who was in Europe between the years 1939–1945, and in Poland in particular, should be defined as a Holocaust survivor, because no matter what the specific nature of the experience was (e.g., concentration camp, hiding in convents or elsewhere) normal life was in jeopardy. Participants in the *comparison group* were in the same age range as that of the Holocaust group, also born in Poland, but immigrated to Israel before the war (before 1939), and were therefore not directly exposed to the Holocaust. We focused on immigrants from Poland because this is the largest community of survivors immigrating to Israel *after 1945,* and of comparisons immigrating to Israel *before 1939*.

This is a retrospective cohort study in which the definition of membership in the two compared groups was retrospectively determined by exposure to the Holocaust (yes or no). Similar to every retrospective study, the current study might have information and selection biases including loss to follow-up. However, the age ranges for the Holocaust and comparison groups were the same, and mean ages (those still alive combined with the estimated age of the deceased) for the Holocaust (M = 85.29 years, SD = 4.35) and the comparison groups (M = 85.57 years, SD = 4.33) were almost identical. This may indicate that there was no differential loss to follow-up in the two groups.

In sum, this population-based retrospective cohort design is derived from the entire population of immigrants from Poland to Israel, 4–20 years old when World War II started, immigrating to Israel either between 1945 and 1950 (Holocaust group) or before 1939 (comparison group; not directly exposed to the Holocaust). Following our approach, potentially confounding factors such as different cultural backgrounds were minimized. We did not include those immigrating during 1940–1944 (actual years of the wars) in order to maximize the likelihood that the group of survivors endured the longest duration of Holocaust experience. Moreover, the similar Polish background was coupled with similar macro life-events and stresses in Israel (e.g., wars, terrorist attacks) in both groups over the years since the declaration of the State of Israel.

It is important to note that deaths among the comparisons should not be registered from an earlier point in time compared to deaths among the Holocaust group. We decided to include in the study only those who were alive at the same date, namely the first day of 1950. At this point in time all the cohort members, Holocaust survivors and comparisons, were in Israel and their deaths have been recorded since in a similar way such that the data are reliable, dependable and comparable. Thus, the ascertainment of death for this study was on a day-to-day basis from 1950 to the end of 2011.

Finally, it should be noted that the minimum age of death in the current data set is 16 years, due to the way death records were created by NII when the system was first established. This is important to acknowledge given that some children in our cohort might have died younger. Such data could not be obtained because during the first years of operation of the Israeli system, it followed the death registry practices of the British administration whereby deaths that took place below the age of 16 were filed in different records than deaths at older ages. No death below the age of 16 is part of the current data base (see Table 1), since our cohorts represent the birth years 1919–1935 and the data set concerns date of death of (alive) subjects as from 1950.

**Table 1:** Description of study cohort – Age of first exposure to Holocaust in 1939.

Total (N = 55,220)	Holocaust (N = 41,454)		Non-Holocaust (N = 13,766)	
	Men*	Women*	Men*	Women*
	20,401 (49.1)	21,053 (50.8)	6,758 (49.1)	7,008 (50.9)
*Age at onset of Holocaust (1939)*				
Childhood (4–9 years)	4112 (49.1)	4,257 (50.9)	1,326 (48.0)	1,438 (52.0)
Adolescence (10–15 years)	7,224 (46.2)	8,408 (53.8)	2,153 (46.2)	2,510 (53.8)
Late Adolescence and Emerging Adulthood (16–20 years)	9,065 (51.9)	8,388 (48.1)	3,279 (51.7)	3,060 (48.3)

* Numbers in brackets are percentages male/female.

### Analyses

We employed a survival analysis using a Cox regression model, which provides estimates of survival probabilities and cumulative hazard, in our case with regards to age of death as related to the Holocaust experience. The hazard of death was the Cox regressed outcome and Holocaust *versus* non-Holocaust the predictive indicator for mapping the regression pattern. The Hazard Ratio (HR) represents the instantaneous risk over the study time period. A HR of 1 means that there is no difference in survival between the two groups. In our study, a HR smaller than 1 means better survival chances for the Holocaust survivors. Analyses included information about death status, namely censored data that refer to participants still alive and event data referring to deceased participants. Because hazard of death is known to be associated with gender [8], we took gender into account both as a control variable and also in interaction with Holocaust status. A significant Gender by Holocaust interaction is followed by a test for specific Holocaust effects for males and females separately. In the present analyses coefficients in the Cox regression imply projection for hazard of death after the Holocaust experience. Survival in months was used in all analyses.

The study cohort had a rather wide age range (4–20 years) at the onset of World War II in 1939, the initial time that exposure to the Holocaust occurred. Therefore we decided to explore in addition whether the hazard ratio was applicable in the same way to subjects of different age groups. We distinguished three age groups according to developmental stage at the start of the Holocaust: childhood (4–9 years), adolescence (10–15 years), and late adolescence/emerging adulthood (16–20 years). Such grouping may shed more light on the hazard ratio along the line of this developmental trajectory.

## Results

**Figure 1:**
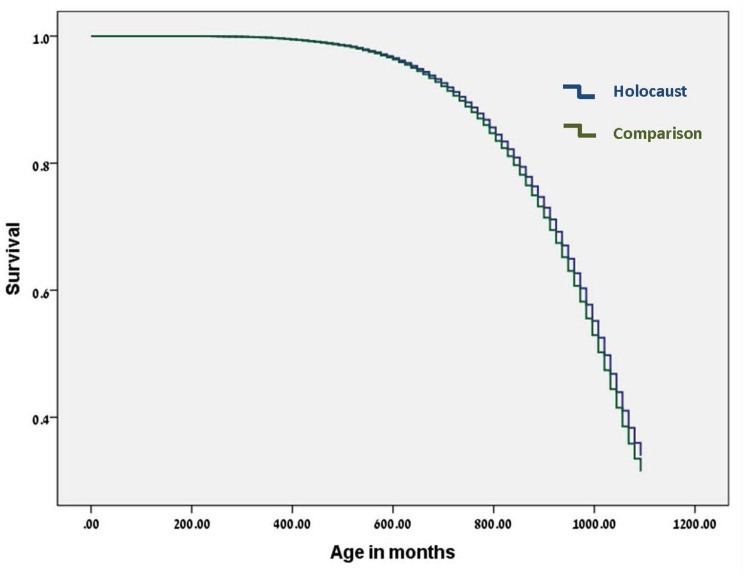
Survival function for the full data set. This figure shows that the hazard of death (instantaneous risk) for Holocaust survivors is significantly smaller than that of non-Holocaust comparisons. The Cox regression indicates that Holocaust survivors’ life expectancy is increased with 6.5 months.

The Cox regression yielded a significant hazard ratio (HR = 0.935, CI (95%) = 0.910–0.960), suggesting that the hazard of death (instantaneous risk) was reduced for Holocaust survivors (exposed group) when compared to non-Holocaust comparisons (non-exposed group) (see Figure 1). Holocaust survivors’ life expectancy was increased with 6.5 months. Gender showed a significant effect as well, with a hazard ratio HR = 1.256, (CI (95%) = 1.224–1.283), implying that males die younger than females.

When gender was used as a stratification variable, the Cox regression yielded a significant hazard ratio (HR = 0.932), CI (95%) = 0.907–0.957) implying once again that the instantaneous risk to die younger was reduced for those exposed to the Holocaust. Because of the significant gender effect, we used this variable not only as a control variable, but also in interaction with Holocaust experience. A significant lower hazard ratio was found (HR = 0.843, CI (95%) = 0.800–0.889), guiding us to examine the hazard of death in Holocaust survivors and comparisons separately for males and females. Only for males did we find that the hazard ratio of Holocaust survivors was reduced. For females it did not make a difference whether they were Holocaust survivors or comparisons.

One assumption of the Cox model is that the hazard ratio remains constant at all times from 1950, the effective beginning of the study. We conducted a series of Cox regressions across the following end points: 1960, 1970, 1980, 1990, 2000, and 2011, which is the end of the study (see Table 2). Our starting point was always 1950, and in the first analysis we used all cases until the end of 1960. The next analysis concerned all cases between 1950–1970, and then 1950–1980, etc., until the final analysis spanned the period to the end of the study, including all cases between 1950–2011. The number of death events by the end of 1960 was only 401 (censored, i.e., still alive *n* = 54,819) and Holocaust exposure did not significantly change hazard. By the end of 1970 the number of death events was 1,314 (censored *n* = 53,906) and again we did not find a significant Holocaust effect. Similarly, by the end of 1980 the number of death events continued to be relatively low, *n* = 3,446 (censored *n* = 51,774) and again no significant Holocaust effect was detected. As can be seen in Table 2, by the end of 1990 significant hazard ratios were observed for Holocaust exposure; the number of death events was more than doubled and then further increased for 2000 and at the end of the study (2011). Across all end points hazard ratios were similar in size and direction, pointing at reduced hazard of death for the group exposed to the Holocaust compared to the non-exposed group.

**Table 2:** Effect of exposure to Holocaust at different end points in time.

End Points (between 1950–2011)	*N*		*Hazard Ratio* *	*95% CI*	*P*
	*Dead*	*Alive*			
**1950–1960**	401	54,819	0.936	0.749–1.170	.561
**1950–1970**	1,314	53,906	0.970	0.857–1.098	.630
**1950–1980**	3,446	51,774	0.991	0.919–1.069	.818
**1950–1990**	7,931	47,289	**0.902**	0.858–0.948	**.000**
**1950–2000**	15,941	39,279	**0.921**	0.889–0.954	**.000**
**1950–2011**	28,479	26,741	**0.932**	0.907–0.957	**.000**

* Gender used as a control variable.

Age of exposure was first treated as a control variable, with Cox regression indicating that, regardless of age, hazard of death for Holocaust survivors was significantly lower (HR = 0.940, CI (95%) = 0.915–0.965). We wanted to explore, though, whether the reduction in hazard was constant across age or whether specific age groups distinctly contributed to the effect. When the analysis was carried out separately for each of the three groups, a significant hazard ratio was found only for the older group of late adolescence/emerging adulthood at the start of the Holocaust (16–20 years) (HR = 0.913, CI (95%) = 0.882–0.946). Because of the substantial gender difference, and in order to obtain more specific outcomes, we then conducted the analyses separately for males and females for each of the three age groups.

As can be seen from the bolded coefficients in Table 3, the Cox regressions for the three age cohorts separately showed a significant effect only for males in the adolescence and late adolescence/emerging adulthood cohorts (i.e., age 10–15 years when first exposed to the Holocaust, HR = 0.900, CI (95%) = 0.842–0.962, and age 16–20 years when first exposed to the Holocaust, HR = 0.820, CI (95%) = 0.782–0.859) suggesting that the general hazard pattern of lower risk of dying at a younger age found in Holocaust survivors than in comparisons is accounted for by the two older male groups. More specifically, the risk of dying is reduced significantly by 10 months and by 18 months respectively for Holocaust survivors who were first exposed to the Holocaust during adolescence or late adolescence/emerging adulthood, as compared to their non-Holocaust counterparts. It can also be seen that the significant effect in the older group is in fact accounted for by the males. Similar to the entire cohort, effects of gender on hazard of death were found across all three age groups, regardless of Holocaust background, showing that the risk of men to die at an earlier age is significantly higher than that of women (Table 4).

**Table 3:** Effect of exposure to Holocaust: Stratified for age and gender.

	*N*		*Hazard Ratio*	*95% CI*	*p*
Age at Exposure (1939)	*Dead*	*Alive*			
**4–9 years**					
*Males*	1,944	3,494	0.991	0.894–1.098	.864
*Females*	1,357	4,338	0.944	0.837–1.065	.350
*Total* *	3,301	7,832	0.971	0.898–1.050	.463
**10–15 years**					
*Males*	4,734	4,643	**0.900**	0.842–0.962	**.002**
*Females*	4,580	6,338	1.061	0.990–1.138	.092
*Total* *	9,314	10,981	0.975	0.928–1.024	.312
**16–20 years**					
*Males*	8,464	3,880	**0.820**	0.782–0.859	**.000**
*Females*	7,400	4,048	1.028	0.977–1.083	.287
*Total* *	15,864	7,928	**0.911**	0.880–0.944	**.000**

* Males and females combined.

**Table 4:** Gender effects across three groups of age at exposure.

	*N*		*Hazard Ratio* **	*95% CI*	*p*
Age at Exposure (1939)	*Dead*	*Alive*			
**4–9 years**	3,301	7,832	**1.651**	1.541–1.770	**.000**
**10–15 years**	9,314	10,981	**1.320**	1.268–1.375	**.000**
**16–20 years**	15,864	7,928	**1.144**	1.109–1.180	**.000**
***Total*** *	28,479	26,741	**1.249**	1.220–1.279	**.000**

* age groups combined.

** Risk of males to die at an earlier age is higher than that of females.

## Discussion

Our results show unexpected outcomes; Holocaust survivors seem to have a significantly lower risk to die younger than comparisons without a Holocaust background. Given that no significant effects were found for the cohorts of children who were first exposed to the Holocaust when they were 4–9 years old, the older cohorts of adolescents as well as late adolescents and emerging adults appear to account for the lower risk of Holocaust subjects to die younger.

As for females, it appears that their usual survival advantage [8] compared to the males sustained itself across all age cohorts, but this advantage was not increased by their Holocaust experiences. By all means and unexpectedly, our findings show no indication of Holocaust survivors being more likely to die at a younger age. If sustained over the entire life-course, the hazard ratios indicate a considerable difference in life expectancy. For the adolescent group, the life expectancy of Holocaust survivors was increased with 10 months, and for the late adolescent and emerging adult group it was increased with 18 months.

Such findings may highlight the resilience of survivors of severe trauma, even when they endured psychological, nutritional, and sanitary adversity, often with exposure to contaminating diseases without accessibility to health services. This may be considered an illustration of the so-called posttraumatic growth [9] that is observed to occur, for example, in soldiers having experienced combat-related trauma but finding greater meaning and satisfaction in their later lives because of those experiences [10] and also in Holocaust child survivors experiencing more social support from friends [11]. An alternative interpretation would be differential mortality, meaning that those vulnerable to life-threatening conditions had an increased risk to die during the Holocaust. Holocaust survivors by definition survived severe trauma, and this may be related to their specific genetic, temperamental, physical, or psychological make-up that enabled them to survive during the Holocaust [12]–[15] and predisposed them to reach a relatively old age. For example, heritability of post-traumatic stress [16] as well as telomere length [17] seems rather large.

The paradox of severe traumatic experiences accompanied by a reduced risk for shorter life expectancy in Holocaust survivors might also be partly explained by the contrast between the precise, objective measure of time of death versus the subjective nature of most parameters of physical and psychological distress used in previous genocide studies involving less reliable self-reports. Furthermore, we report here on the largest Holocaust study that has ever been conducted. Because of the large number of subjects included in our study there is no issue of insufficient statistical power concerning statistical analyses. Moreover, relying on the NII registry precludes issues of recruitment of participants through convenience or potentially biased samples (e.g. mental health clinics, Holocaust-related organizations, advertisements) as is the case with many Holocaust studies.

A number of limitations should be noted. First, albeit not dying at a younger age, Holocaust survivors may have experienced lower quality of life, for example, higher rates of invalidity or non-fatal illnesses. Second, we have no data available on factors determining death rates during the war, and differential mortality remains an interpretation in need of empirical evidence. Third, one may also contend that those who emigrated before the Holocaust had in some way a weaker health and were liable to die at a younger age. One of the main motivations for emigration at such an early stage was however “Zionistic” in nature, namely, to go to the “old Jewish land” with the desire to cultivate it, as well as a desire of many young people to live an adventurous life. [18] It is therefore speculative but reasonable to argue that these were not the less healthy individuals. Fourth, one may similarly pose the hypothesis that the stronger persons who survived the Holocaust chose to stay in Europe or leave Europe to the USA (as many did) and that only the weaker had no choice but to go Israel, in which case Holocaust survivors might be at greater hazard for shorter life expectancy. However, the data show otherwise, which may preclude this bias. One should also recall that during 1945–1948, many of the Holocaust survivors were not allowed to enter the British Mandate of Palestine (pre-state of Israel). Thus it could be the case that we have here a bias in the opposite direction because the stronger individuals might have been more successful in fighting their way to Palestine whereas the weaker stayed in Europe. Of course all these suggestions remain speculations that should be kept in mind. It should be noted that later emigration out of Israel has not been sufficiently documented by NII. The Cox regression assumes that the population is dynamic and it considers the hazard on the basis of person years since exposure. Ideally, emigrated subjects would be censored at the date of emigration but no systematic registry of such data exists. The present analyses are based on the assumption that emigration out of Israel of the two groups was similar, but of course there is room for potentially differential emigration of which we are not aware. Lastly, our data were derived from government records without information obtained in direct contact with the participants, and therefore we have no data about type of experience during the Holocaust or about biological markers of aging such as telomere length, so we cannot address potential links between various experiences and longevity. Such information might have shed further light on our findings. Regardless of their experiences, however, Holocaust survivors were not found to live shorter than their comparisons but in fact seemed to have a likelihood of living even *longer* than their counterparts without Holocaust background.

In sum, meta-analytic evidence from numerous studies [5] comparing Holocaust survivors with comparisons documents strong post-traumatic stress reactions even more than half a century after the end of World War II. But the current epidemiological study failed to find a lower life expectancy for Holocaust survivors, and even found higher age expectancies for Holocaust survivors in the male cohorts who were adolescents or late adolescents/emerging adults when first exposed to the Holocaust. Elder [19] studied children exposed to the Great Depression and found that they followed a trajectory of resilience into the middle years of life that was most noticeably among the oldest group (adolescents) as compared with younger children. Elder’s seminal work may suggest that the more “positive” effect found among the older Holocaust survivors is perhaps associated with the fact that those who were in their adolescence or emerging adulthood at the onset of war displayed more physical strength and resilience as compared to younger and more vulnerable children.

Our remarkable findings of the relative longevity of Holocaust survivors by no means imply any extenuation of the immense trauma caused by the Holocaust and by a sad succession of other genocides. On the contrary, our findings highlight the importance of public health policies providing socio-emotional and medical support for individuals who managed to survive atrocious circumstances.
